# Parallel graded attention in reading: A pupillometric study

**DOI:** 10.1038/s41598-018-22138-7

**Published:** 2018-02-27

**Authors:** Joshua Snell, Sebastiaan Mathôt, Jonathan Mirault, Jonathan Grainger

**Affiliations:** 1 Aix-Marseille Université, Marseille, France; 2 Brain & Language Research Institute, Aix-en-Provence, France; 3 Rijksuniversiteit Groningen, Groningen, The Netherlands; 4 Centre National de Recherche Scientifique, Marseille, France

## Abstract

There are roughly two lines of theory to account for recent evidence that word processing is influenced by adjacent orthographic information. One line assumes that multiple words can be processed simultaneously through a parallel graded distribution of visuo-spatial attention. The other line assumes that attention is strictly directed to single words, but that letter detectors are connected to both foveal and parafoveal feature detectors, as such driving parafoveal-foveal integrative effects. Putting these two accounts to the test, we build on recent research showing that the pupil responds to the brightness of *covertly attended* (i.e., without looking) locations in the visual field. Experiment 1 showed that foveal target word processing was facilitated by related parafoveal flanking words when these were positioned to the left and right of the target, but not when these were positioned above and below the target. Perfectly in line with this asymmetry, in Experiment 2 we found that the pupil size was contingent with the brightness of the locations of horizontally but not vertically aligned flankers, indicating that attentional resources were allocated to those words involved in the parafoveal-on-foveal effect. We conclude that orthographic parafoveal-on-foveal effects are driven by parallel graded attention.

## Introduction

One of the most hotly debated issues in reading research concerns the question of whether words are processed serially or in parallel^1–5^. Of particular relevance in this debate are the recent lines of research pointing out that the recognition of words in the fovea is influenced by parafoveal orthographic information. The principal finding is that words are recognized faster when they are orthographically related to surrounding words or letters. For instance, several studies have found the fixation duration on word *n* to be decreased when it was orthographically related to *n* + 1 during sentence reading^1–5^. Furthermore, in the flanker paradigm, lexical decisions about isolated target words were found to be faster and more accurate when those targets were flanked by related letters on each side (e.g. ‘*ro rock ck*’) as compared to unrelated letters (e.g. ‘*st rock ep*’)^4–6^.

The conception that foveal word processing is influenced by surrounding words might lead one to conclude that attentional resources must have been allocated to the foveal word and surrounding words in parallel^7–12^. According to the parallel processing approach, the attentional distribution would follow a Gaussian shape centered on the attentional focus^9^, such that while processing of the fixated word would normally be the strongest, surrounding words and letters may nonetheless exert some influence, hence explaining the so-called parafoveal-on-foveal effects listed above.

Yet, these effects are not considered by all to provide conclusive evidence that attention is distributed across multiple words. Indeed, one alternative theory proposes that visuo-spatial attention is strictly directed to one word at a time (i.e. serial processing accounts of reading)^13^, but that foveal letter detectors would be connected to both foveal and parafoveal feature detectors. Parafoveal orthographic information would as such influence foveal letter processing without having received any attentional resources^3^. Importantly however, syntactic and semantic variants of the flanker paradigm have shown that syntactic decisions (e.g., *noun/verb*) and semantic decisions (e.g., *natural/artifactual object*) about foveal target words are made faster if those targets were flanked by congruent words as compared to incongruent words (stimulus on-time 170 ms)^7,8^. Crucially, there was no orthographic overlap between targets and flankers, implying that the idea of parafoveal feature detectors influencing foveal letter detectors cannot account for these particular findings. It must be noted however, that while these higher-order (e.g. syntactic, semantic) parafoveal-on-foveal effects show up in artificial reading tasks such as the flanker paradigm, they are not expressed in more natural measures of reading speed, such as fixation durations in sentence reading^3,7,8^ ‒ possibly because higher-order information is simply not integrated across words during normal reading.

The debate of serial versus parallel processing still ongoing, it is apparent that the field is in dire need of a more direct measure of the allocation of attention during reading. This is precisely what we aim to provide with the present work. Specifically, we report a novel methodology that builds on recently obtained evidence that the pupillary light response can reflect visual attention.

## Using the pupil to track attention

The pupillary light response consists of a constriction of the pupil in brightness (to reduce the amount of light entering the eye) and a dilation of the pupil in darkness (to maximize the amount of light entering the eye). It was historically considered to be a low-level reflex without any cognitive component^14^. However, in recent years it has become clear that pupil size changes may reflect a multitude of higher-order phenomena, such as awareness^15^, interpretation^16^ and mental imagery^17–19^.

Concerning attention, in a paradigm where participants responded to target stimuli appearing in the left- or right visual hemifield, with the screen background being vertically split into a white and a black half with a luminance-neutral (gray) band in the middle, it was found that presenting an auditory cue prior to target onset (‘left’ or ‘right’, indicating the probable location of the target) caused the pupil to dilate more if the cued side was black, as compared to if the cued side was white, indicating that the shift of covert (i.e., without looking) visual attention triggered a pupillary light response^20^. Crucially, the eye position was carefully tracked, so as to ensure that the overall amount of light entering the pupil was equal across conditions.

A more recently implemented paradigm showed that the pupil size could reveal which one out of several parafoveal orthographic stimuli participants covertly attended^21^. In this paradigm, packaged as a ‘mind-writing’ interface, eight letters were presented in a circle around a central fixation point. Each letter was presented on a background that oscillated between white and black, with four letters being on a black background during the time that the other four letters were on a white background, and vice versa. Attending one of the letters (while continuing to focus on the screen center) caused the pupil size to oscillate in cadence with the background of that letter, thus allowing the computer to bring the amount of possibly attended letters from eight to four. These four letters were then again divided into two groups with opposing background brightness, until the pupil size led the computer to select two candidate letters. A final repetition of this procedure led the computer to deduce the truly attended letter.

## Involving pupillometry in the flanker paradigm

It is clear then, that the pupil size is contingent with the brightness of covertly attended locations in the visual field. This sparks a straightforward prediction concerning the role of attention in parafoveal-on-foveal effects: if attentional resources are indeed allocated to parafoveal stimuli, then the pupil size should be influenced by the brightness of the locations of these stimuli (keeping the overall screen brightness equal). To this end we devised a series of experiments wherein we could obtain varying degrees of parafoveal-foveal integration, while manipulating the brightness of parafoveal locations. The flanker paradigm was particularly suited for this, as it simulates the conditions of reading in a controlled manner that does not necessitate the reader to make saccades, while nevertheless allowing us to determine to which extent readers are engaged in additional processing by surrounding words. The aim was to see, firstly, whether pupil size would be affected by the brightness of flanker locations, and secondly, whether this pupil size effect would covary with the degree of parafoveal-foveal integration.

In order to obtain varying degrees of parafoveal-foveal integration, we hypothesized that flankers positioned above and below the target word should have a smaller impact on target processing than flankers positioned left and right of the target, given that attention is mainly distributed along the horizontal axis during reading (in scripts that are aligned horizontally)^22^. Experiment 1 tested this hypothesis by letting participants make lexical decisions about foveal target words, while orthographically related or unrelated words were presented either left and right of the target, or above and below the target (stimulus on-time 150 ms).

All experiments were carried out with approval of the Scientific and Ethical committee of the Aix-Marseille University, and were in accordance with the declaration of Helsinki. All data gathered for this work are publicly available at https://osf.io/jm938/.

## Experiment 1: A first test of horizontal vs. vertical integration

### Method

#### Participants

Twenty students from Aix-Marseille University gave informed consent to participate in this study, carried out at the Laboratoire de Psychologie Cognitive (Marseille, France) for €4,- or its equivalent in course credit. All participants were native to the French language, non-dyslexic, and naïve to the purpose of the study.

#### Materials

We retrieved a list of 80 4-letter target words from the French Lexicon Project database (Ferrand *et al*.^23^). These targets were noun or non-conjugated verb and contained no diacritics (e.g. *é, ô, ç*). Each target was paired up with a 4-letter control word that met the same criteria as the target and that shared no letters with the target. The average frequency of targets and control words was equal, at 5.41 Zipf (a log10-based frequency measure)^24^.

In a similar fashion we retrieved 80 4-letter non-word targets from the French Lexicon Project pseudoword database^23^, and paired each of these with a non-word control. The non-word targets were used as filler stimuli and were not included in the data analyses.

#### Design

**Figure 1:**
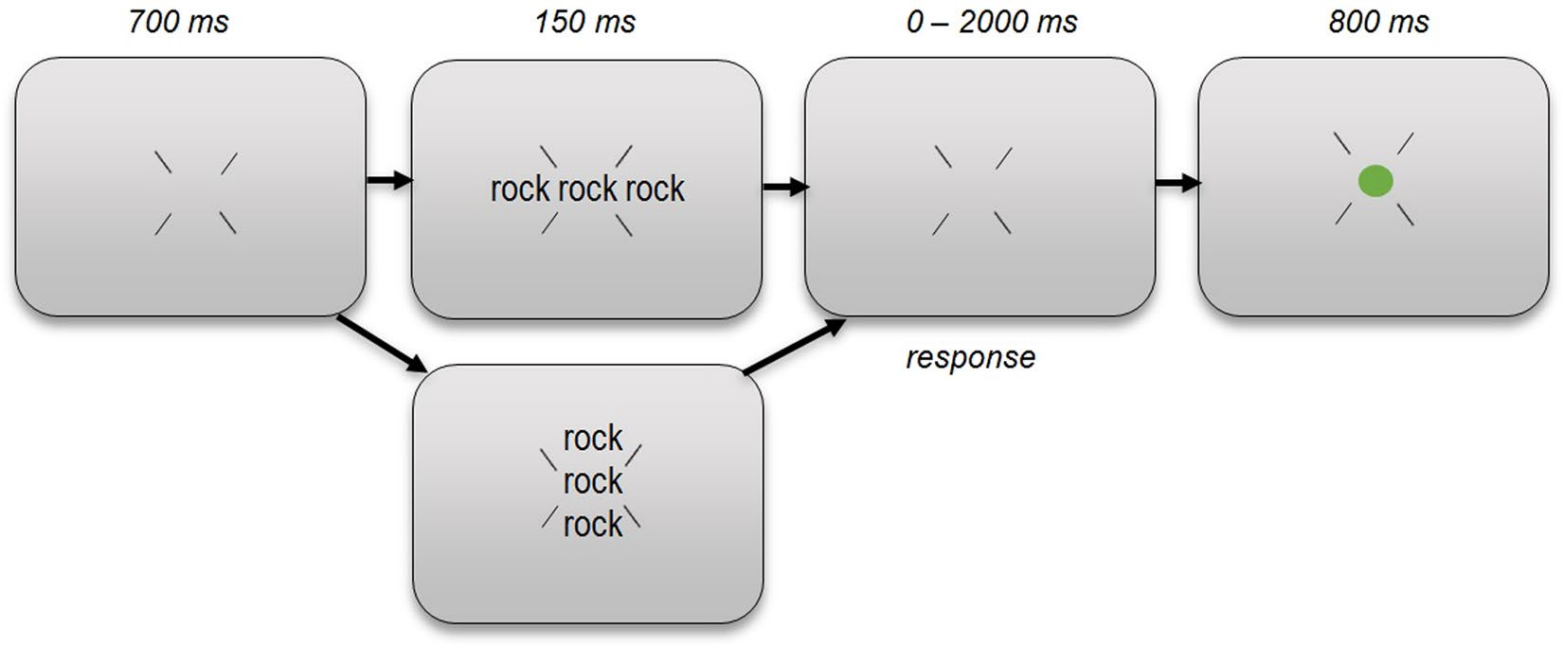
Experiment 1 trial procedure. After a 700 ms fixation display, the target was presented for 150 ms, flanked either by the same word (shown here) or the unrelated control word, left and right of the target (top panel) or above and below the target (bottom panel). Participants then had a maximum of 2000 ms to respond whether the target was a word or non-word, after which feedback was provided (green or red dot for correct and incorrect answers respectively). Note that this figure only shows two of the four possible target displays. The size of stimuli relative to the display is exaggerated in this figure.

Experiment 1 followed a 2 × 2 factorial design with flanker relatedness (related/unrelated to the target) and flanker position (above and below the target/left and right of the target) as factors (note that the target lexicality factor is disregarded here). In the related flanker conditions, the target word was repeated at the flanker locations (Fig. 1). In the unrelated conditions, the control word was shown at the flanker locations. Participants were Latin-squared into two groups to ensure that all stimuli were shown in all four conditions, but only twice per participant (with the same flanker shown in both flanker positions). The experiment thus consisted of 320 trials (including non-word trials), and these were presented in random order.

#### Apparatus and software

The experiment was implemented with OpenSesame^25^ and presented on a 1024 × 768 px, 150 Hz computer monitor. Participants were seated in a comfortable office chair at a distance of 50 cm from the display, so that each character space subtended 0.40 degrees of visual angle. Responses were collected with a gamepad (using two trigger buttons for the left and right index finger) at a polling rate of approximately 125 Hz.

#### Procedure

Participants received instructions both verbally by the experimenter and visually onscreen. Participants were instructed to maintain their focus at the center of the display, guided by four diagonally oriented fixation bars (Fig. 1). 700 ms after the start of each trial, the target was presented for 150 ms (in line with previous implementations of the flanker paradigm)^4–6^, together with two flankers that were either the same word or the unrelated control word. These flankers were positioned left and right of the target, or above and below the target, with the word centers at a distance of 90 pixels from the screen center. After the target and flankers disappeared, participants had a maximum of 2000 ms to respond whether the target was a word or non-word, with a right- or left-sided button press respectively. Feedback was provided after each response (green or red dot for correct and incorrect answers, respectively). The experimental trials were preceded by a block of eight practice trials, and participants were offered a break halfway through the experiment. The experiment lasted approximately 20 minutes.

### Results

For the analysis of response times (RTs) we included all correctly answered trials (with a word target) for which the RT was no further than 2.5 standard deviations from the grand mean (86.91% of trials). The latter criterion led us to exclude 2.12% of trials for the analysis of error rates.

For the RT analysis we used a linear mixed-effects model (LMM) with items and participants as crossed random effects^26^. We determined the maximal random effects structure permitted by the data, leading us to include the interaction of flanker relatedness × position as by-item and by-participant random slopes alongside random intercepts^27^. We report regression coefficients (*b*), standard errors (SE) and *t-*values, with values |*t*| >1.96 deemed significant^26^. A logistic LMM was used to analyze the error rates, (here we report *z-*values instead of *t*-values). In this particular model, a failure to converge under the maximal random effects structure led us to include only the by-item and by-participant random intercepts. The models were fitted with the lme4 package^28^ in the R statistical computing environment.

Table 1 shows the mean RTs and error rates for all conditions. There was a significant main effect of flanker relatedness (Table 2), with unrelated flankers leading to longer RTs than related flankers. There were also significantly more errors in the unrelated flanker conditions. Meanwhile, there was no main effect of flanker position.

**Table 1:** Experiment 1 condition means.

Flanker type	RTs (ms)		Error rates	
	related	unrelated	related	unrelated
Left/right of target	403.94 (114.37)	443.33 (122.33)	0.08 (0.06)	0.15 (0.06)
Above/below target	403.26 (120.81)	404.93 (113.60)	0.08 (0.06)	0.09 (0.05)

Note: values in between parentheses indicate standard deviations.

**Table 2:** Analyses of RTs and error rates (ref.: left/right related flankers).

	RTs			Error rates		
	b	SE	t	b	SE	z
(intercept)	404.69	13.95	**29.01**	2.75	0.20	13.99
Relatedness (R)	42.48	7.35	**5.78**	0.77	0.17	**4.63**
Position (P)	0.37	6.51	0.06	0.04	0.18	0.20
R × P	40.58	9.79	**4.14**	0.73	0.25	**2.95**

Note: significant values are shown in bold.

In line with our hypothesis, there was a significant interaction of flanker relatedness and flanker position (Table 2). As can be clearly seen in Table 1, the effect of flanker relatedness was strongly expressed for the left- and right-positioned flankers (Λ ≈ 40 ms), while it was virtually absent when flankers were positioned above and below the target.

### Discussion

Our aim with Experiment 1 was to see whether various degrees of parafoveal-foveal integration could be obtained using horizontally vs. vertically aligned stimuli. We predicted that flankers positioned to the left and right of the target would have a stronger impact on target processing than flankers positioned above and below the target, in line with the idea that attention would be mainly distributed along the horizontal axis during reading. This hypothesis was confirmed, as we found a strong effect of flanker relatedness with horizontally aligned stimuli but not with vertically aligned stimuli (Table 1).

Although these results support the conception that parafoveal-on-foveal effects are driven by parallel graded attention, they do not provide conclusive evidence. Within the alternative line of reasoning – i.e., that foveal letter detectors may be connected to parafoveal feature detectors (causing parafoveal information to have an impact without the necessity of being attended)^3^ – it is possible that the letter detectors are mainly connected to parafoveal feature detectors in the horizontal dimension rather than the vertical dimension. This would in turn lead to an influence of words to the left and right of fixation, but not of words above and below fixation.

Following the rationale outlined in the Introduction, if the parafoveal-on-foveal effects of horizontally aligned flankers were driven by parallel graded attention, then the reader’s pupil size should be contingent with the brightness of the locations of those flankers. In contrast, the pupil size should not be contingent with the brightness of flankers that did not impact on target processing—that is, the vertically aligned flankers.

This prediction was put to the test in Experiment 2. In a setting similar to that of Experiment 1, we manipulated the brightness of flanker locations (i.e., flankers had either a black or white background), and hypothesized a flanker brightness × position interaction effect on the pupil size, with black flanker backgrounds causing a dilated pupil compared to white flanker backgrounds, specifically for horizontally but not vertically aligned flankers. Masks (‘####’) were presented at the flanker positions perpendicular to that of the word flankers, with a background color opposite to that of the word flankers, so that the overall luminance of the display was equal across conditions. The eye position and pupil size were tracked during a fixed 2450 ms interval, allowing us to assess the pupillary light response in full. As in Experiment 1, targets, flankers and masks were shown for 150 ms on each trial, while the flanker backgrounds were kept onscreen throughout the 2450 ms interval.

## Experiment 2: Flanker brightness and the pupillary light response

### Method

#### Participants

Twenty-four students from the Aix-Marseille University gave informed consent to participate in this study, carried out at the Laboratoire de Psychologie Cognitive (Marseille, France) for €5,- or its equivalent in course credit. All participants were native to the French language, non-dyslexic and naïve to the purpose of the study. Further, all participants reported to have normal vision (and thus did not use glasses or contact lenses, which tend to disturb the eye-tracker signal).

#### Materials

As we extended the experimental design with an additional factor (flanker brightness), we increased the total amount of target words (and non-word targets) to 100. The selection criteria were left unchanged from Experiment 1. The average frequency of targets and control words was 5.40 and 5.46 Zipf, respectively.

#### Design

Experiment 2 followed a 2 × 2 × 2 design, with flanker relatedness (related/unrelated to the target), position (left and right of the target/above and below the target) and background brightness (white/black flanker background) as factors. Participants were Latin-squared into four groups, such that all targets were shown across all conditions, but only twice per participant (with the same flanker identity and position shown in both the dark and the bright background setting). The experiment thus totaled 400 trials (including non-word trials), and these were presented in random order.

#### Apparatus

The PyGaze back-end^29^ was used to process eye movement data online. The participant’s right eye position was recorded with an EyeLink 1000 (SR Research, Mississauga, ON, Canada), a video-based eye tracker sampling at 1000 Hz with a spatial resolution of 0.01°. We acknowledge that the tracking of a single eye prevented us from taking a potential degree of binocular disparity (which may have induced minor noise in the data) into account. Participants were seated at a 90 cm distance from the display, so that each character space subtended 0.35 degrees of visual angle. A chin-rest was used to facilitate a stable head position. Responses were collected with a keyboard instead of a gamepad this time.

#### Procedure

**Figure 2:**
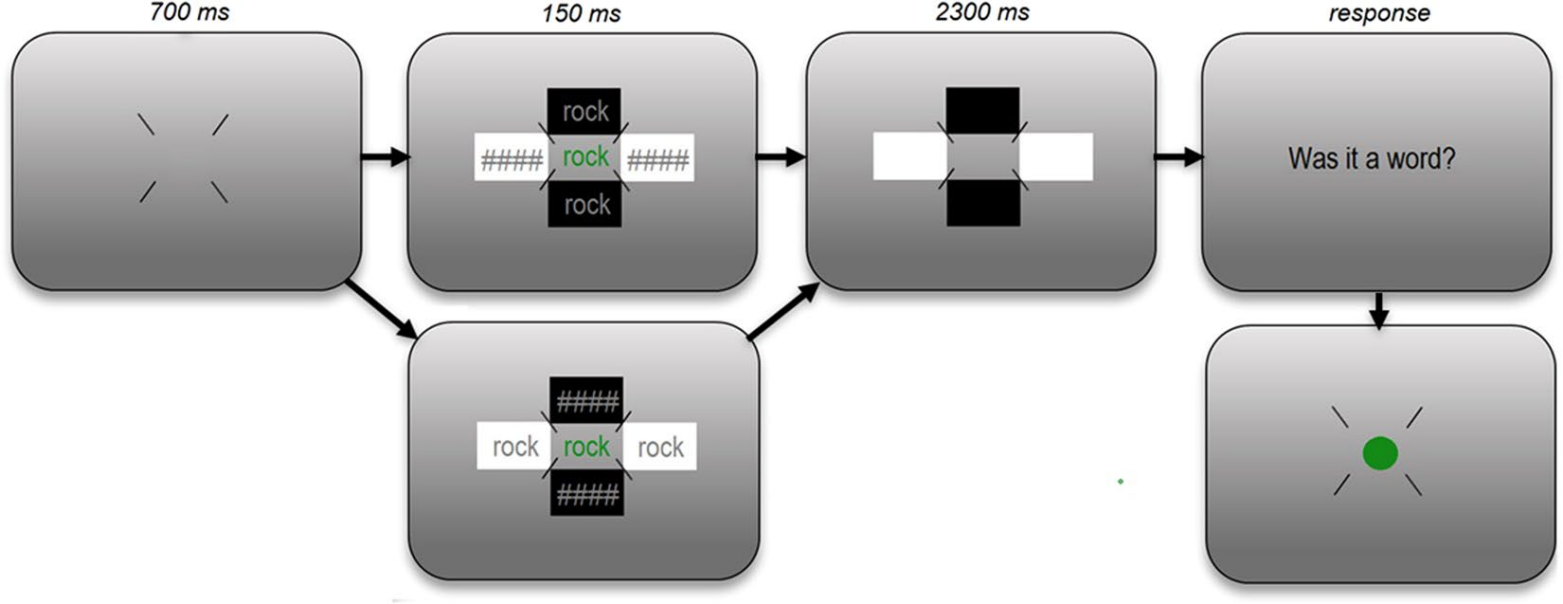
Experiment 2 trial procedure. The target was presented in a luminance-neutral green color on a gray background. Flankers were presented in luminance-neutral gray on a white or black square-shaped background, with masks being presented on backgrounds of the opposite color. Note that this figure only shows two of the eight possible target displays. The size of stimuli relative to the screen is exaggerated in this example.

Prior to the start of the experiment, the participant’s right eye position was calibrated using a 9-point calibration grid. The trial display (Fig. 2) differed from that of Experiment 1 in two respects: firstly, the flankers were presented in luminance-neutral (gray) color on either a white or black square background, while the target was presented in luminance-neutral green. Secondly, gray masks (‘####’) were shown at the flanker positions perpendicular to the word flanker positions, with a background color opposite to that of the word flankers, to ensure that the overall display luminance was equal across conditions.

Participants were instructed to maintain their focus at the screen center. As in Experiment 1, targets and flankers were presented for 150 ms. Unlike Experiment 1 however, in Experiment 2 participants were instructed to provide their response during a response display that was presented 2300 ms after the target- and flanker offset. As can be seen in Fig. 2, the flanker backgrounds stayed onscreen throughout this interval. The pupil size was measured during 2450 ms after stimulus onset, thus allowing us to assess the pupillary light response in full. Note that participants were not instructed to respond as fast as possible in this experiment because the response itself influences the pupil size; a difference in RT between conditions would as such lead to a cofounded pupil size effect. Participants were offered a break halfway through the experiment. The experiment lasted approximately 45 minutes.

### Results

Since participants were instructed only to respond when the response display was presented (2300 ms after stimulus offset), we do not report RTs for Experiment 2. The error rates are presented in Table 3. In line with previous results, significantly more errors were made in the unrelated flanker conditions compared to the related flanker conditions (*b* = 0.61, SE = 0.23, *z* = 2.61), but this effect was not modulated by flanker position (*b* = 0.32, SE = 0.33, *z* = 0.96).

**Table 3:** Experiment 2 error rates.

Flanker type	Related	Unrelated
Left/right of target	0.02 (0.02)	0.04 (0.04)
Above/below target	0.03 (0.03)	0.04 (0.02)

Note: values in between parentheses indicate standard deviations.

#### Pupil size

Prior to the analyses of pupil size, the pupil size was normalized and baseline-corrected (taking the average of 300 ms prior to stimulus onset) such that the pupil size at stimulus onset was equal to 0. The data was downsampled by factor 10 (i.e., taking the average of every 10 ms of data), such that each 2450 ms interval was represented by 245 datapoints per subject. An LMM with flanker brightness, flanker position and flanker relatedness as factors and subjects and items as crossed random effects was run over the course of 245 cycles, each representing 10 ms of pupil size data.

**Figure 3:**
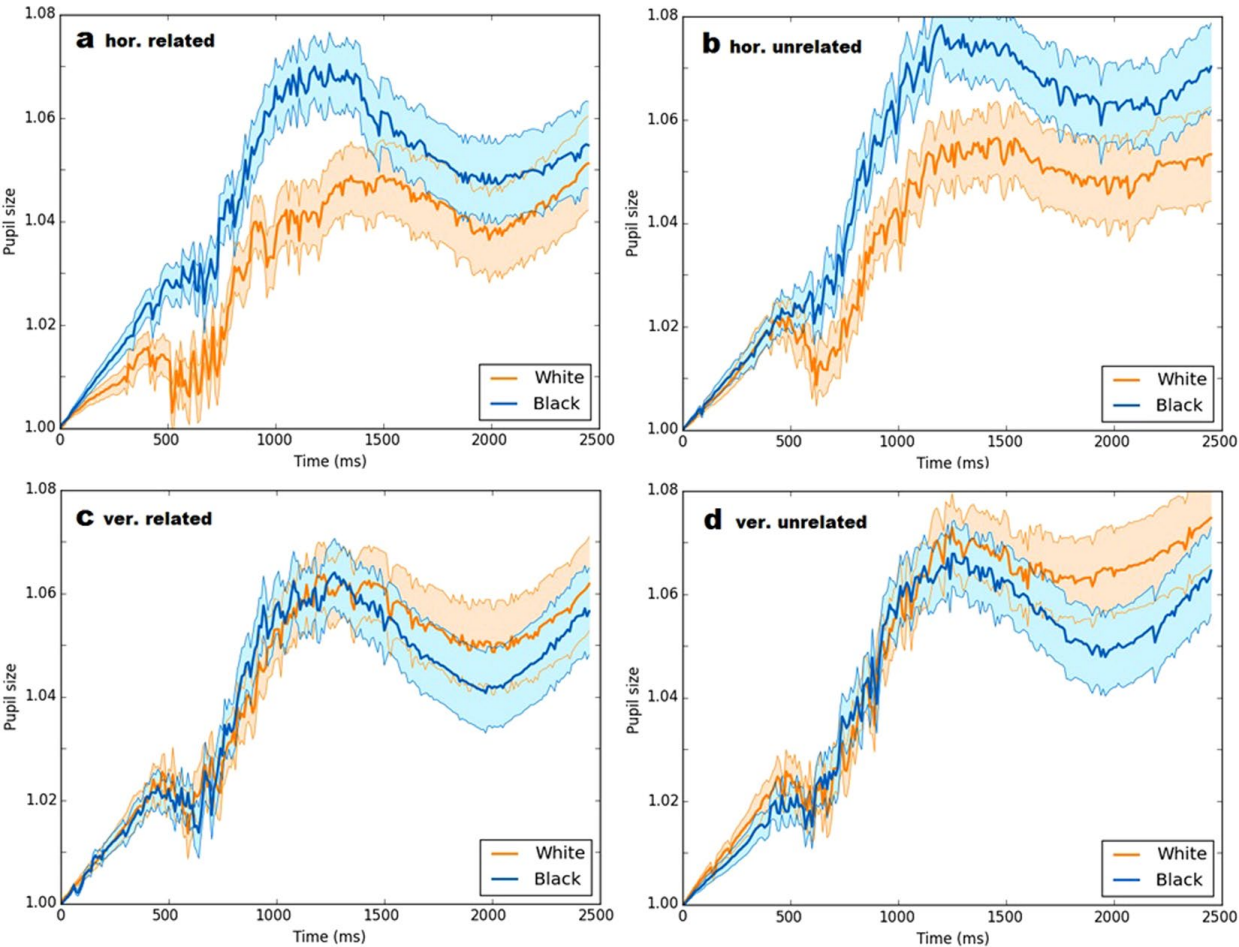
Average pupil size during trials in which the word flankers had black backgrounds (blue lines) versus white backgrounds (orange lines), when the flankers and target were horizontally aligned (panels a and b) and vertically aligned (panels c and d) respectively. Panels (a) and (c) show the related flanker conditions, whereas panels (b) and (d) show the unrelated flanker conditions. The blue and orange shaded areas indicate standard errors.

Figure 3 shows pupil size deflections for horizontally aligned flankers (top two panels) and vertically aligned flankers (bottom two panels), both in the related conditions (left panels) as well as the unrelated conditions (right panels). It is apparent that the pupil size was contingent with the location brightness of horizontally aligned flankers, given the decreased pupil size in the presence of a white background compared to a black background in these conditions (top two panels). In contrast, the pupil size deflection was quite similar for white and black background flankers when these were vertically aligned (bottom two panels).

**Figure 4:**
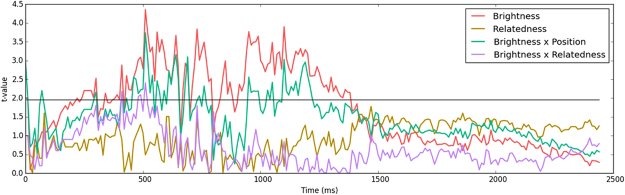
Experiment 2 analysis outcomes. The y-axis represents statistical significance, with the significance threshold (|*t*| = 1.96) being indicated by the black horizontal line. The analysis was carried out on each 10 ms time step, amounting to a total of 245 analyses for the 2450 ms interval.

As can be seen in Fig. 4, a significant main effect of flanker location brightness was established around the 250 ms mark (with marginal significance showing as early as 200 ms after stimulus onset). Crucially, we also established a significant interaction of flanker location brightness and flanker position, such that the pupil size was contingent with the brightness of horizontally but not vertically aligned flankers, in line with our hypothesis. It is apparent that the data is auto-correlating: i.e., intervals of significance are preceded and followed by trends towards significance, indicating that the observed effects are no false positives (which are by default likely to occur in multiple-comparison analyses).

Interestingly, a significant interaction of flanker location brightness and flanker relatedness emerged around the 500 ms mark, such that the effect of location brightness was enhanced if the flankers were orthographically related to the target. Post-hoc analyses revealed that this effect was driven by the conditions with horizontally aligned word flankers, as the pattern persisted when viewing these conditions in isolation, but not when viewing vertically aligned word flanker trials in isolation. Neither a main effect of flanker relatedness (Fig. 4), nor a three-way interaction of location brightness, relatedness and position was established.

### Discussion

In Experiment 1 we found that information is integrated across horizontally but not vertically aligned stimuli. Crucially, as established in Experiment 2, this asymmetry is also expressed in the pupil size – i.e., we obtained a pupil size effect with horizontally aligned flankers, but not with vertically aligned flankers, indicating that attention was directed specifically to the locations of those stimuli involved in the orthographic parafoveal-on-foveal effect. The flanker location brightness effect started manifesting itself as early as 200 ms after stimulus onset (Fig. 4), which is the fastest latency associated with the pupillary light response^14^. This immediacy of effects thus suggests that a portion of attention was directed to the flanking stimuli *during* – rather than *after* – processing of the target, in line with accounts of reading that assume a parallel graded distribution of attention.

We further established an interaction of flanker location brightness and flanker relatedness around the 500 ms mark. Post-hoc analyses (not reported above) revealed that this effect persisted when analyzing horizontally aligned word flanker trials in isolation, but not when analyzing vertically aligned word flanker trials in isolation. This particular finding is quite remarkable in that it reflects how the reading process is driven by a continuous interaction of various cognitive levels. Specifically, parafoveal orthographic processing led to stronger lexical activation in the related flanker conditions, subsequently leading to enhanced low-level visual processing and as such an increased pupillary light response.

## General Discussion

In this paper we have presented a novel methodology to track the distribution of visuo-spatial attention in a controlled reading setting. This methodology was employed to address a key phenomenon driving the debate about whether words are processed serially or in parallel during reading: namely, orthographic parafoveal-on-foveal effects. The principal finding is that words are recognized slower when surrounded by orthographically unrelated information (e.g., ‘*st rock ep’*) compared to related information (‘*ro rock ck*’). At present, two lines of theory exist to account for these effects. One line assumes that visuo-spatial attention is allocated to multiple words in parallel, as such allowing for the integration of information across the fovea and parafovea^4,5,7–9^. The other line assumes that attention is strictly allocated to single words, but that letter detectors are connected to both foveal and parafoveal feature detectors, as such causing word processing to be influenced by parafoveal orthographic information^3^.

To provide a direct test of the theory that attention can be allocated to multiple words in parallel, we made use of the principle that the pupil responds to the brightness of covertly attended locations in the visual field^19–21^. We predicted that if orthographic parafoveal-on-foveal effects are indeed driven by parallel distributed attention, then the pupil size should be contingent with the brightness of those stimuli that are involved in the parafoveal-on-foveal effect. We therefore aimed to create a setting in which we could obtain various degrees of parafoveal-foveal integration while manipulating the brightness of parafoveal stimuli – expecting the latter to cause a pupil size effect specifically in those conditions that produced an orthographic parafoveal-on-foveal effect.

In Experiment 1 we found that foveal word processing was impacted by horizontally but not vertically aligned adjacent words, as lexical decisions about foveal target words were slowed and less accurate when those targets were flanked by orthographically unrelated words on the left and right (compared to being flanked by a repetition), but not when the same word was presented above and below the target. Following the above rationale, we thus hypothesized that a manipulation of the brightness of flanker locations would cause a pupil size effect with horizontally aligned flankers, but not with vertically aligned flankers.

The results of Experiment 2 were perfectly in line with this hypothesis: an interaction of flanker location brightness and flanker position was established, such that the pupil size was affected by the brightness of words located left and right of the target, but not words located above and below the target. Of crucial importance here is that the pupil size effect started manifesting itself as early as 200 ms after stimulus onset (Fig. 4), which is the minimal latency associated with the pupillary light response^14^, thus indicating immediate processing whereby portions of attention were allocated to the parafoveal words *during*, rather than *after*, target processing.

Remarkably, the pupillary light response was modulated by the orthographic relatedness of flankers as well – albeit only for a short time window approximately 500 ms after stimulus onset. Our account of this particular finding is that increased lexical activation due to integration of orthographically related information in turn leads to increased activation in earlier visual areas through recurrent processing, consequently enhancing the pupillary light response. It must be acknowledged however, that this interpretation of effects is as of yet merely speculative.

A question that remains, is whether the present findings speak to the reading system in general, and sentence reading in particular. Indeed, one could argue that attention may be strictly directed to single words during sentence reading, while it would be distributed across multiple words in ‘unnatural’ reading settings such as the experiments reported in this paper. Undoubtedly these settings differ in nature, as is attested by the fact that higher-order (e.g. syntactic, semantic) parafoveal-on-foveal integration was observed in the flanker paradigm^7,8^ but not in sentence reading^3,7,8^ (however, see^30^). It is possible, however, that such differences are driven by how the reading system organizes incoming information in each respective setting, rather than by how attention is distributed. Indeed, given that readers are not able to effectively focus attention on single words in the flanker paradigm, it stands to question how readers could then manage to do so during sentence reading, considering that (i) parafoveal words are more relevant and important during sentence reading, (ii) parafoveal words are available longer during sentence reading (compared to 150 ms in the flanker paradigm), and (iii) the visual input during sentence reading is more complex and dynamic due to eye-movements. It may in this light be more sensible that the reading system in principle processes multiple words at once, but that mechanisms driving sentence-level comprehension prevent cross-leakage of higher-order information between words during sentence reading; (see^7,8^ for a detailed discussion of this possibility).

In sum, the present results suggest that orthographic parafoveal-on-foveal effects are driven by parallel processing of multiple words through a widespread distribution of visuo-spatial attention. We conclude that the pupillary light response is a fruitful means to addressing the role of attention in reading.

## References

[1] Inhoff, A., Radach, R., Starr, M. & Greenberg, S. Allocation of visuospatial attention and saccade programming during reading in *Reading as a perceptual process* (eds Kennedy, A., Radach, R., Heller, D. & Pynte, J.) 221–246 (Elsevier, 2013).

[2] Vitu F, Brysbaert M, Lancelin D (2004). A test of parafoveal-on-foveal effects with pairs of orthographically related words. European Journal of Cognitive Psychology.

[3] Angele B, Tran R, Rayner K (2013). Parafoveal–foveal overlap can facilitate ongoing word identification during reading: Evidence from eye movements. Journal of Experimental Psychology: Human Perception and Performance.

[4] Dare N, Shillcock R (2013). Serial and parallel processing in reading: Investigating the effects of parafoveal orthographic information on nonisolated word recognition. The Quarterly Journal of Experimental Psychology.

[5] Snell J, Vitu F, Grainger J (2017). Spatial integration of parafoveal orthographic information: Beyond the sub-lexical level?. Quarterly Journal of Experimental Psychology.

[6] Grainger J, Mathôt S, Vitu F (2014). Test of a model of multi-word reading: Effects of parafoveal flanking letters on foveal word recognition. Acta Psychologica.

[7] Snell J, Meeter M, Grainger J (2017). Evidence for simultaneous syntactic processing of multiple words during reading. PLoS ONE.

[8] Snell, J., Declerck, M. & Grainger, J. Parallel semantic processing in reading revisited: Effects of translation equivalents in bilingual readers. *Language, Cognition and Neuroscience*., 10.1080/23273798.2017.1392583 (in press).

[9] Engbert R, Nuthmann A, Richter M, Kliegl R (2005). SWIFT: A dynamical model of saccade generation during reading. Psychological Review.

[10] Kennedy A, Pynte J (2005). Parafoveal-on-foveal effects in normal reading. Vision Research.

[11] Reilly R, Radach R (2006). Some empirical tests of an interactive activation model of eye movement control in reading. Cognitive Systems Research.

[12] Engbert, R. & Kliegl, R. Parallel graded attention models of reading in *The Oxford Handbook of* Eye *Movements* (eds. Liversedge, S., Gilchrist, I. & Everling, S.) 787–800 (Oxford University Press, 2011).

[13] Reichle E, Liversedge S, Pollatsek A, Rayner K (2009). Encoding multiple words simultaneously in reading is implausible. Trends in Cognitive Science.

[14] Loewenfeld I (1958). Mechanisms of reflex dilatation of the pupil. Documenta Ophthalmologica.

[15] Naber M, Frassle S, Einhauser W (2011). Perceptual rivalry: Reflexes reveal the gradual nature of visual awareness. PLoS ONE.

[16] Binda, P., Pereverzeva, M. & Murray, S. Pupil constrictions to photographs of the sun. *Journal of Vision* **13**, 10.1167/13.6.8 (2013). 10.1167/13.6.8 23685391

[17] Laeng B, Sulutvedt U (2014). The eye pupil adjusts to imaginary light. Psychological Science.

[18] Mathôt S, Strijkers K, Grainger J (2017). Pupillary responses to words that convey a sense of brightness or darkness. Psychological Science.

[19] Mathôt S, van der Stigchel S (2015). New light on the mind’s eye: The pupillary light response as active vision. Current Directions in Psychological Science.

[20] Mathôt S, van der Linden L, Grainger J, Vitu F (2013). The pupillary response to light reflects the focus of covert visual attention. PLoS ONE.

[21] Mathôt S, Melmi J, van der Linden L, van der Stigchel S (2016). The mind-writing pupil: A human-computer interface based on decoding of attention through pupillometry. PLoS ONE.

[22] Rayner K (1998). Eye movements in reading and information processing: 20 years of research. Psychological Bulletin.

[23] Ferrand L (2010). The French Lexicon Project: Lexical decision data for 38,840 French words and 38,840 pseudowords. Behavior Research Methods.

[24] Van Heuven W, Mandera P, Keuleers E, Brysbaert M (2014). SUBTLEX-UK: A new and improved word frequency database for British English. The Quarterly Journal of Experimental Psychology.

[25] Mathôt S, Schreij D, Theeuwes J (2012). OpenSesame: An open-source, graphical experiment builder for the social sciences. Behavior Research Methods.

[26] Baayen, R. *Analyzing Linguistic Data: A pratical introduction to statistics*. Cambridge: Cambridge University Press (2008).

[27] Barr D, Levy R, Scheepers C, Tily H (2013). Random effects structure for confirmatory hypothesis testing: Keep it maximal. Journal of Memory and Language.

[28] Bates D, Maechler M, Bolker B, Walker S (2015). Fitting Linear Mixed-Effects Models usinglme4. Journal of Statistical Software.

[29] Dalmaijer E, Mathôt S, Van der Stigchel S (2014). PyGaze: An open-source, crossplatform toolbox for minimal-effort programming of eyetracking experiments. Behavior Research Methods.

[30] Snell J, Grainger J (2017). The sentence superiority effect revisited. Cognition.

